# The demographic features and outcome indicators of the Barbados HIV Pre-exposure Prophylaxis Program, 2018-2019

**DOI:** 10.26633/RPSP.2021.51

**Published:** 2021-04-23

**Authors:** Anton Best, Nastassia Rambarran

**Affiliations:** 1 Ministry of Health and Wellness Bridgetown Barbados Ministry of Health and Wellness, Bridgetown, Barbados; 2 Independent researcher Independent researcher

**Keywords:** HIV, pre-exposure prophylaxis, Barbados, Caribbean Region, VIH, profilaxis pre-exposición, Barbados, Región del Caribe, HIV, profilaxia pré-exposição, Barbados, Região do Caribe

## Abstract

**Objective.:**

To assess the demographics, risk profiles and outcome indicators of one of the few government-supported programs on pre-exposure prophylaxis (PrEP) in the English-speaking Caribbean.

**Methods.:**

Chart review of all persons enrolled into the national PrEP Program from its inception on March 1^st^ 2018 to November 30^th^ 2019, with a descriptive summary analysis of the data extracted.

**Results.:**

Of the 134 persons enrolled into the program most identified as men who have sex with men (67.9%), followed by men who have sex with men and women (14.9%); there were 20 persons, mostly men (85%), in sero-discordant relationships. PrEP uptake was 96%; however, the continuation rate (continuing for three consecutive months after initiation) was 61.5%. Continuation status for many could not be ascertained due to loss-to-follow-up. PrEP-associated toxicity prevalence was 2.3%, although side-effects occurred in 52% (mostly gastrointestinal). HIV positivity during the study period was 1.5%.

**Conclusions.:**

Uptake of Barbados’ national PrEP Program is excellent but fairly low continuation rates and the HIV positivity rate indicate the need for improved pre-ART initiation education and follow-up processes. Service utilisation is mainly by men who have sex with men, and provision expansion to other civil society partners and private practitioners, as well as increased public awareness could increase access by other high-risk groups.

## RESUMEN

**Objetivos.:**

Evaluar la información demográfica, los perfiles de riesgo y los indicadores de resultados de uno de los pocos programas de profilaxis previa a la exposición (PrEP, por su sigla en inglés) respaldado por un gobierno en el Caribe de habla inglesa.

**Métodos.:**

Análisis de gráficos de todas las personas que se registraron en el Programa Nacional de PrEP desde su inicio el 1 de marzo de 2018 hasta el 30 de noviembre 2019, con un análisis de los datos extraídos en forma de resumen descriptivo.

**Resultados.:**

De las 134 personas que se registraron en el programa, la mayoría se identificaba como hombres que tienen relaciones sexuales con hombres (67,9%), seguido de hombres que tienen relaciones sexuales con otros hombres y con mujeres (14,9%). Había 20 personas, principalmente hombres (85%), en relaciones serodiscordantes. La tasa de captación del programa de PrEP fue de 96%; sin embargo, la tasa de continuación (continuar en el programa durante tres meses consecutivos después del inicio) fue de 61,5%. En muchos casos no se pudo verificar la continuación debido a la falta de seguimiento. La prevalencia de la toxicidad asociada con la PrEP fue de 2,3%, aunque se observaron efectos colaterales en un 52% (principalmente en el aparato digestivo) de las personas. La positividad para el VIH durante el período del estudio fue de 1,5%.

**Conclusiones.:**

El Programa Nacional de PrEP de Barbados tiene una tasa de captación excelente; sin embargo, la tasa de continuación relativamente baja y la tasa de positividad para el VIH indican la necesidad de mejorar la educación inicial previa al tratamiento antirretroviral y los procesos de seguimiento. Estos servicios fueron utilizados principalmente por hombres que tienen relaciones sexuales con hombres; si se ampliase el suministro de estos a otros colaboradores de la sociedad civil y médicos particulares, y si se concientizase más al público, se mejoraría el acceso para otros grupos de alto riesgo.

## RESUMO

**Objetivos.:**

Avaliar as características demográficas, perfis de risco e indicadores de resultados de um dos poucos programas de profilaxia pré-exposição (PrEP) com apoio governamental existentes em países de língua inglesa do Caribe.

**Métodos.:**

Revisão dos prontuários de todos os participantes inscritos no Programa Nacional de PrEP desde o seu início em 1. de março de 2018 a 30 de novembro de 2019, com uma análise sumária descritiva dos dados extraídos.

**Resultados.:**

Dos 134 participantes inscritos no programa, a maioria se identificou como homens que fazem sexo com homens (67,9%), seguido de homens que fazem sexo com homens e mulheres (14,9%). Vinte participantes, sobretudo homens (85%), estavam em relacionamentos sorodiscordantes. O índice de captação de participantes ao PrEP foi de 96%, porém a taxa de continuidade no programa (continuar participando por três meses consecutivos após ter iniciado o programa) foi de 61,5%. Não foi possível confirmar a continuidade no programa em muitos casos por perda de seguimento. A prevalência de toxicidade associada à PrEP foi 2,3%, apesar de terem ocorrido efeitos colaterais em 52% dos participantes (na sua maioria efeitos gastrointestinais). A taxa de positividade ao HIV durante o período de estudo foi de 1,5%.

**Conclusões.:**

A captação do Programa Nacional de PrEP de Barbados foi excelente, mas a continuidade da participação relativamente baixa e a taxa de positividade ao HIV observada indicam que é necessário instruir melhor as pessoas antes de iniciar a terapia antirretroviral e aprimorar o processo de acompanhamento. Este programa é utilizado principalmente por homens que fazem sexo com homens. Ampliar a prestação do serviço a outros parceiros da sociedade civil e a profissionais particulares, assim como melhorar a conscientização do público, poderia aumentar o acesso por outros grupos de alto risco.

Daily oral HIV pre-exposure prophylaxis (PrEP) with tenofovir disoproxil fumarate /emtricitabine (TDF/FTC) has been proven effective and safe in high-risk groups, which prompted the World Health Organization (WHO) to recommend it as an additional HIV prevention choice for these vulnerable groups (1). Although PrEP access and national programs have increased since 2016, over 500 000 or 75% of persons utilizing the intervention are within the African continent and United States, and the global target of providing 3 million at-risk persons with PrEP by 2020 was still unreached by mid-2020 (2-4). Apart from Brazil, where almost 30 000 persons are on PrEP, only 8 other Latin America and Caribbean countries have started enrolling small numbers (less than 5 000 total) due to challenging regulations, high price points and lack of awareness by both providers and potential clients (1, 4). In the Caribbean, the barriers to utilizing PrEP center around cost, lack of awareness, and concerns about risk compensation among policy makers and health care providers (5-7), although interest in PrEP has been high in key population communities of men who have sex with men (MSM), transgender persons and sex workers who have been made aware of the option (8, 9).

Barbados is a small independent nation in the Caribbean where the HIV prevalence rate among the general population aged 15 to 49 years is 1.5%, with a higher prevalence of 11.8% in MSM (10). Ninety percent of persons living with HIV know their status, although only 52% are on treatment, and 46% are virally suppressed (11). Treatment occurs at the national treatment centre, the Ladymeade Reference Unit (LRU), complemented by a few private medical practitioners, government polyclinics and civil society organizations (CSOs). Pertinent legislative context includes the fact that Barbados still criminalises anal sex with up to life imprisonment (12) and does not constitutionally prohibit discrimination on the grounds of sex, sexual orientation or gender identity (13). However, progressive steps include the recent Employment Act which prevents discrimination on the basis of sex and sexual orientation (14), a national gender policy under review (13), and that employers cannot require HIV testing or discriminate against persons living with HIV (15).The HIV prevalence data, along with sustained high incidence and prevalence of other sexually transmitted infections (STIs) in the country, prompted the Barbados Ministry of Health and Wellness to add PrEP to its combination prevention approach to HIV in March 2018. Barbados is the second country in the English-speaking Caribbean, after the Bahamas, to have implemented PrEP at a national level, although there are other countries in the region, such as Guyana, Grenada and Jamaica, where implementation is planned or PrEP is offered in a very limited manner to certain populations (16, 17). The Barbados guidelines for PrEP were developed based on guidance from the WHO and the US Centers for Disease Control and Prevention (CDC) and state that PrEP may be offered to any person in the country who is deemed to be at substantial risk for HIV (18). The eligibility criteria include adults over 18 years who are HIV negative with no suspicion of acute HIV infection and at least one of the following in the previous 6 months: ongoing sexual relationship with an HIV-positive partner who is not virally supressed; being MSM or transgender and engaging in unprotected anal sex; having unprotected transactional sex; being MSM, transgender or a sex worker with an STI; having condom-less sex with partners of unknown HIV status who are at substantial risk of HIV infection; and having had post-exposure prophylaxis for sexual exposure. The guidelines also note that individuals who do not fit into the aforementioned risk categories may qualify or request PrEP based on perceived risk of exposure and that decisions to initiate should be individualized by weighing HIV risk against the potential benefits and risks of TDF/FTC (18). The intervention is offered for free and utilised daily oral administration, with on-demand PrEP being extended to a few select clients at the end of the study period. There were several forms of generic TDF/FTC available for dispensation and the guidelines dictated follow-up appointments three months after initiation of PrEP, then at least every three months after that depending on comorbidities, risk behaviour and laboratory results that would warrant more frequent monitoring (18). PrEP in Barbados was initially only available at LRU, but expanded to the lesbian, gay, bisexual and transgender plus (LGBT+) CSO Equals in February 2019, via a “Shared Care” system with the HIV/STI Programme of the Ministry of Health and Wellness (19). Locally, persons received official PrEP information from Equals, LRU, and other CSOs and private doctors who completed PrEP training. Anyone desirous of starting PrEP made an appointment and underwent individual or couple’s pre-initiation counselling on medication use, effectiveness and side-effects by a medical doctor. This counselling was sometimes supplemented by a nurse or peer educator who was often the first point of contact. At this consultation, a screening questionnaire and brief medical history were also administered. If eligible and ready, blood for HIV rapid testing using a sequential algorithm beginning with the Alere Determine^®^ kit, confirmatory HIV testing, syphilis (VDRL), hepatitis B/C, HTLV 1 and 2, liver and kidney functions, and urine for PCR chlamydia and gonorrhoea testing was taken. After a negative rapid test, persons were offered PrEP and uplifted tablets right away at LRU but had up to a one-week wait at Equals. They were advised to contact the site/doctor with any queries or reports of severe side effects before their 3-month appointment.

This study aimed to provide an assessment of the national program, specifically the demographic composition and risk profiles of the enrolled, along with core outcome indicators during the first two years of program rollout. This will help to provide empirical evidence to adjust and guide the Barbados national program, and be useful for other countries where comparable population and HIV epidemic dynamics are at play, and policy makers await information from similar settings to inform their PrEP implementation.

## MATERIALS AND METHODS

The study utilised a quantitative methodology involving a review of the medical records of all persons enrolled into the Barbados national HIV PrEP program between March 1st 2018 and November 30th 2019. Persons who might have expressed an interest in PrEP or been told about PrEP, but not officially enrolled in the program by having a PrEP registration number assigned and a PrEP medical file created were excluded from the review. Data was collected from the records, anonymously entered into a Microsoft Excel^®^ spreadsheet and the analyses were conducted using the analytical software SPSS v.26 (IBM Corp, Armonk, NY). These consisted of simple descriptive analyses, calculation of core indicators (uptake, continuation, toxicity and positivity) as suggested by the WHO (20), and statistical analysis among demographic features, risk factors and outcomes using Chi-square, Fisher exact test, and Student’s t-test. P-values were considered statistically significant at <0.05.

This study was approved by the UWI/ Barbados Ministry of Health Research Ethics Committee/Institutional Review Board (IRB No. 200102-A).

## RESULTS

One hundred and thirty-four (134) persons were enrolled into the program during the study period. The majority were cisgender men (94%), with 6 cisgender women and 2 transgender women. Clients averaged 30 years old, with most identifying as MSM (67.9%), followed by men who have sex with men and women (MSMW) (14.9%); there were 20 persons in sero-discordant relationships: 9 men who have sex with women (45.0%), 8 MSM (40.0%) and 3 women who have sex with men (15.0%). The majority were Barbadian (85%), with most of the other nationalities coming from the Caribbean region, and an almost equal number of persons had enrolled at either site (50.7% at Equals and 49.3% at LRU). Most persons had no co-morbidities (60.0%), but among those who did, asthma was the most common (47.1%), followed by hypertension (15.0%).

Upon enrolment, 20.8% of persons reported previous STIs, commonly syphilis (67.8%), followed by chlamydia (25.0%) and gonorrhea (25.0%). When tested on enrolment, there were 18 cases of reactive titers indicating infection with syphilis (13.4%), 3 cases of chlamydia (2.2%), 2 of gonorrhea (1.5%) and 1 case of HTLV-1 infection (0.7%), for a total of 24 prevalent infections (17.9%). A notable number of persons reported that they always used condoms during the 6 months prior to enrolment (23.8%). Most had had more than three partners in the preceding 6 months (34.4%), whilst others had two partners (31.3%), one partner (29.8%) or none (4.6%). Six (6) persons did not commence anti-retrovirals (ARVs) for PrEP after enrolment due to likely allergy, deferral of decision or comorbidities that prevented the administration of the medication.

During monitoring while on PrEP, only 2 persons reported symptoms of STIs, but screening tests revealed 4 new syphilis infections, 2 new chlamydia infections and 1 case of hepatitis C. There were minor elevations of liver enzymes, none reaching the threshold for discontinuation of PrEP, and only one patient had a creatinine clearance of less than 60 mL/min. Side effects occurred in 52% of patients, with some patients reporting multiple side effects. They were mostly gastrointestinal in nature (nausea, vomiting, bloating, gas, diarrhea, flatulence, abdominal cramps) (74.0% of persons with side effects), although headaches (14.2% of persons with side effects) and sleep disturbances (insomnia, vivid dreams) (14.2% of persons with side effects) were also commonly reported. There were no serious adverse events, but one person with a history of multiple allergies discontinued the medication after a possible allergic reaction. Amongst persons who had at least one follow-up visit and were asked about change in their condom use since starting PrEP (40/67), most reported no change (60.0%), whilst others used condoms less (25.0%) or more (10.0%). Some pointed out that condoms were only used less with regular partners or because they had a mild latex allergy. Persons missed an average of 3 pills in the previous 30 days, mostly because they forgot.

PrEP uptake was based on the WHO definition (20), as the percentage of eligible people who initiated the intervention (starting ARVs for PrEP) within the study period and calculated as 95.5%. The WHO definition for the continuation rate as the number of PrEP users who continued on the medication for three consecutive months after having initiated it, was calculated as 63.1%; continuation status for many could not be ascertained due to being lost to follow-up. Table 1 shows the disaggregation of uptake, continuation and lost to follow-up rates according to age, gender identity and risk category and reveals that although the numbers are small, cisgender women had 100% uptake and continuation rates while men who have sex with women had the lowest continuation rates. PrEP associated toxicity prevalence, which is defined by WHO as the percentage of people who received PrEP but discontinued it due to serious medication-associated toxicity, was 2.3% while the percentage of people who tested HIV-positive among people who received PrEP at least once during the study period and had at least one follow-up HIV test (WHO HIV positivity rate) was 1.5% (1/64). This person had defaulted from follow-up for almost a year, and by the time it was noticed and they were called into clinic for reassessment, they had seroconverted to an HIV positive status.

Analysis of characteristics associated with continued enrolment (defined as not discontinued or been lost to follow-up) by the end of the study period showed that more persons remained enrolled at Equals (75%) than LRU. Being lost to follow-up – defined as having missed an appointment by over 2 months with no reason noted in the records – was significantly associated with enrolment at LRU (93.1%, *p* < 0.00). Persons who continued enrolment were likely to be slightly older (31.3 years, SD 11.5) compared to those who had discontinued or been lost to follow-up (29.5 years, SD 6.8), *t* (101), *p* = 0.02. Having three or more partners within the 6 months prior to enrolment was associated with continued enrolment (73.2%) when compared to persons who had one (44.8%) or two partners (6.2%) over that same period (*p* < 0.02). Interestingly, those who reported always using condoms in the 6 months before enrolment were also more likely to have continued enrolment (81.5%) versus those who did not always use condoms (50%) (*p* < 0.00). Nationality, risk category (MSM, MSMW, etc.) or the presence of any side effects were not correlated with continued enrolment in the program as can be seen in Table 2 where the analysis on categorical variables is presented. Since only 15 reasons for discontinuation were recorded, an analysis of this data was not conducted, but such reasons included migration, not being sexually active, medication side effects, settling with one partner, and being “no longer interested”.

**TABLE 1.** Uptake, continuation and lost to follow-up rates by age, gender and risk category, Barbados HIV Pre-exposure Profphylaxis Program, 2018-2019

Demographic	Uptake (%)	Continuation (%)	Lost to follow-up (%)
**Age**			
<25	33/36 (91.6)	17/26 (65.3)	4/9 (44.4)
25-44	85/87 (97.7)	42/69 (60.8)	17/27 (62.9)
>44	10/11 (90.9)	6/8 (75)	2/2 (100)
**Gender**			
Cis women	6/6 (100)	5/5 (100)	0
Trans women	2/2 (100)	1/2 (50)	0
Cis men	120/126 (95.2)	59/96 (61.4)	22/37 (59.4)
**Risk category**			
Women who have sex with men	8/8 (100)	6/7 (85.7)	0
Men who have sex with men	87/91 (95.6)	44/68 (64.7)	12/24 (50)
Men who have sex with men and women	20/20 (100)	12/17 (70.5)	2/5 (40)
Men who have sex with women	12/12 (100)	3/10 (30)	3/7 (42.8)

## DISCUSSION

The efficacy of PrEP has been conclusively shown in clinical trials, such as the iPrEx, IPERGAY and Partners PrEP, but reports from real-world implementation have only been available for around five years and largely focused on demonstration projects which are smaller scale research endeavours designed to assess acceptability, cost and program design for scale-up (21, 22). This study is the first of its kind in the English-speaking Caribbean and while the uptake rate is narrowly defined as those who initiated PrEP from among those formally assessed for eligibility (as opposed to anyone who had enquired about PrEP or been told about the intervention, which was impossible to ascertain), the excellent rate of uptake demonstrates the widescale acceptability of PrEP in Barbados, which has a largely Black population of African descent, and in both men and women. This finding is unsurprising given that the Barbados Ministry of Health and Wellness has reported anecdotally high demand for PrEP (19). Supporting data for this demand is scant, but one unpublished online survey of 188 persons (of whom 85% were MSM), showed modest demand of 52% of persons not on PrEP being interested in the intervention (23). In Brazil, a demonstration project also showed similarly high uptake (96%) in persons who attended enrolment visits (24).This is in opposition to uptake rates in Black American MSMs where uptake is low, with up to 84% reduced odds compared to white participants in one study, due to social and structural barriers such as access, cost, perceived side effects and the influence of social and sexual networks (25). Having an HIV-positive partner, greater risk behaviour (multiple partners and condom-less sex) and worrying about HIV have been found to be associated with increased PrEP uptake (26), although this study did not assess factors associated with uptake due to the small numbers of those who did not initiate PrEP. Other studies have used different metrics to investigate continuation or retention rates and these have varied from 0 to 90% depending on the population and time frame (21). Among Kenyan MSM, 22% of persons remained on PrEP at 3 months (27), while 71% of young American MSM (28), 74.3% of Australian persons (mostly MSM) (29), and 83% of Brazilian MSM and transgender women stayed on PrEP (30) in various studies. The low continuation rates in this study reflect the need for increased systematic capacity to conduct follow-ups to determine barriers to continuation, as well as the need for better pre-initiation counselling. Especially low continuation rates among men who have sex with women, many of whom were in sero-discordant relationships, may be due to better viral suppression in the HIV positive partners or discontinuation of the sero-discordant partnership, which improved clinical follow-up would be better able to ascertain. Initial trials showed disappointing retention and adherence rates in women, but subsequent demonstration projects have revealed a reversal of these findings, although most of these projects were conducted among female sex workers (22, 27). This study enrolled a very small number of women, but the good uptake and continuation rates hold promise for future engagements with women who could benefit from PrEP and the necessary expansion of the national program.

**TABLE 2.** Analysis of factors associated with continued enrolment, Barbados HIV Pre-exposure Profphylaxis Program, 2018-2019

Characteristic	Percentage that continued enrolment	Chi-square p value
**Site enrolment**		
Enrolled at Equals	75.0	0.00
Enrolled at LRU	41.8	
**Risk category**		
Women who have sex with men	100.0	0.16
Men who have sex with men	56.0	
Men who have sex with men and women	60.0	
Men who have sex with women	60.0	
**Number of partners in last 6 months**		
0	33.3	<0.02
1	44.8	
2	46.2	
3 or more	73.2	
**Side effects**		
No side effects	100.0	0.4
Had side effects	74.2	
**Nationality**		
Barbadian	59.3	0.42
Non-Barbadian	47.1	
**Condom use in last 6 months**		
Always	81.5	0.00
Not always	50.0	

Other studies have presented adverse events as a graded measure based on clinical trials, and did not use the WHO toxicity prevalence as specifically calculated in this study; however, the low toxicity prevalence in this study aligns with the low rates of reported grade 2 to 4 events in other studies (28-30). Approximately half of the clients in this study reported side effects which they linked to the ARV use. The predominance of gastrointestinal issues is common across the literature, but this rate is higher than that reported in trials and in Australian and Indian demonstration projects, where side effects occurred in around one third of persons (22, 29, 31). The discrepancy may be explained by reported side-effects being actually unrelated to ARV use and recall bias given the length of time between initiation and follow up (3 months). This high rate of side effects did not translate to high discontinuation rates among those followed up, in keeping with their transient nature. HIV positivity rates during PrEP use are generally low (29, 30, 32, 33), and the one case of sero-conversion in this program (who was not on PrEP at the time of HIV acquisition, but had been lost to follow-up from the PrEP program), underscores the necessity for continued follow-up and continuation of ARVs in high-risk clients.

The high prevalence of STIs on enrolment and continued incidence of infection during monitoring show the value of PrEP as a component of the combination prevention approach. Syphilis was the most commonly detected STI, continuing the trend of elevated infection rates following an outbreak that occurred in Barbados in 2011 (34), and mirroring similarly high levels in Brazil; syphilis increases HIV acquisition two to nine-fold (30), thus highlighting the need to screen for, and treat STIs. Change in condom use after starting PrEP was the only measured indicator on risk compensation, with the majority of persons demonstrating no risk compensation. This adds to the currently conflicting evidence on whether, and by how much, risk compensation occurs in MSM (30). The use of a key population-led CSO to assist in delivery of PrEP has been a successful strategy elsewhere (21, 22), and this holds true for Barbados as evidenced by high rates of uptake and continued enrolment at Equals. Equals employed a healthcare provider dedicated to PrEP provision, ensuring that there were few persons lost to follow-up, in contrast to LRU where the providers had multiple other job responsibilities. Results from similar country-wide programs are not yet widely available, but in Australia rapid and sustained uptake was noted in men and the 25% discontinuing PrEP were more likely to be female, younger and receiving government benefits (35). In Brazil’s program, 97% of the discontinuations were persons lost to follow-up, who were also more likely to be women, younger, and undomiciled (36). In both Kenya and the Bahamas, partnerships with communities were critical to national initiation (37, 38). In the Bahamas, where the government program has been operating since 2016, roll-out has been slow, with low continuation rates, highlighting the importance of assessing PrEP readiness, provider awareness, and decreasing stigma (37). The results from Australia, Brazil and the Bahamas, along with our findings, reinforce the need for pre-initiation counselling that pays special attention to the demographics most likely to discontinue PrEP.

### LIMITATIONS

This study is limited by its small sample size and sparse enrolment of women and transgender persons, thus preventing in-depth analysis by gender. Use of self-reporting, which is prone to social desirability and recall biases, decreased the robustness of analysis related to indicators on behaviour and adherence, and the absence of uniform recordings of changes in partner numbers post-enrolment, difference in condom use, substance use and sex work precluded proper investigation of these variables. The large number of persons lost to follow-up by the end of the study period meant that the toxicity prevalence could possibly be higher than calculated, but it’s unlikely the HIV positivity rate is also higher given that almost every newly diagnosed case of HIV comes to the attention of LRU and would have been flagged as a previous PrEP patient in the system (assuming that they became HIV+ and were diagnosed).

## CONCLUSION

This program evaluation presents important lessons for similar design and patient counselling for HIV and STI programs in the region. Whilst the excellent uptake rate (96%), low toxicity prevalence (2.3%), HIV positivity (1.5%) and risk compensation are positive trends, the low continuation rates (61.5%), high loss to follow-up and mostly MSM client base highlight needed improvements in program design, including better pre-initiation counselling, follow-up procedures and possibly task-shifting with expanded capacities or the designation of personnel solely responsible for PrEP provision, as was practiced at Equals, This could accompany the decentralisation of PrEP provision to more private providers and CSOs providing sexual and reproductive health services, enabling the integration of PrEP into other health care services and expansion of clientele to other high-risk populations, especially women. Increased public sensitization on the availability and role of PrEP will be necessary to expand the service and decrease any stigmatization of PrEP as key population-specific, but will need to be balanced with the cost of expansion. Additional and uniform recording procedures will allow further future delineation of factors associated with continuation, adherence and behaviours, and should be accompanied by a qualitative component for more detailed insight into contexts and influences.

## Disclaimer.

Authors hold sole responsibility for the views expressed in the manuscript, which may not necessarily reflect the opinion or policy of the RPSP/PAJPH and/or PAHO.
